# CD4 T lymphocyte autophagy is upregulated in the salivary glands of primary Sjögren’s syndrome patients and correlates with focus score and disease activity

**DOI:** 10.1186/s13075-017-1385-y

**Published:** 2017-07-25

**Authors:** Cristiano Alessandri, Francesco Ciccia, Roberta Priori, Elisa Astorri, Giuliana Guggino, Riccardo Alessandro, Aroldo Rizzo, Fabrizio Conti, Antonina Minniti, Cristiana Barbati, Marta Vomero, Monica Pendolino, Annacarla Finucci, Elena Ortona, Tania Colasanti, Marina Pierdominici, Walter Malorni, Giovanni Triolo, Guido Valesini

**Affiliations:** 1 Dipartimento di Medicina Interna e Specialità Mediche, Sapienza Università di Roma, Rome, Italy; 2 Dipartimento Biomedico di Medicina Interna e Specialistica, Università degli Studi di Palermo, Palermo, Italy; 3 Dipartimento di Biopatologia e Biotecnologie Mediche e Forensi, Università di Palermo, Palermo, Italy; 4 Pathology Unit, Azienda Ospedaliera Ospedali Riuniti Villa Sofia-Cervello, Palermo, Italy; 5 Centro per la Medicina di Genere, Istituto Superiore di Sanità, Rome, Italy

**Keywords:** Sjögren syndrome, Autophagy, Lymphocytes, Cytokines

## Abstract

**Background:**

Primary Sjögren’s syndrome (pSS) is a common chronic autoimmune disease characterized by lymphocytic infiltration of exocrine glands and peripheral lymphocyte perturbation. In the current study, we aimed to investigate the possible pathogenic implication of autophagy in T lymphocytes in patients with pSS.

**Methods:**

Thirty consecutive pSS patients were recruited together with 20 patients affected by sicca syndrome and/or chronic sialoadenitis and 30 healthy controls. Disease activity and damage were evaluated according to SS disease activity index, EULAR SS disease activity index, and SS disease damage index. T lymphocytes were analyzed for the expression of autophagy-specific markers by biochemical, molecular, and histological assays in peripheral blood and labial gland biopsies. Serum interleukin (IL)-23 and IL-21 levels were quantified by enzyme-linked immunosorbent assay.

**Results:**

Our study provides evidence for the first time that autophagy is upregulated in CD4^+^ T lymphocyte salivary glands from pSS patients. Furthermore, a statistically significant correlation was detected between lymphocyte autophagy levels, disease activity, and damage indexes. We also found a positive correlation between autophagy enhancement and the increased salivary gland expression of IL-21 and IL-23, providing a further link between innate and adaptive immune responses in pSS.

**Conclusions:**

These findings suggest that CD4^+^ T lymphocyte autophagy could play a key role in pSS pathogenesis. Additionally, our data highlight the potential exploitation of T cell autophagy as a biomarker of disease activity and provide new ground to verify the therapeutic implications of autophagy as an innovative drug target in pSS.

**Electronic supplementary material:**

The online version of this article (doi:10.1186/s13075-017-1385-y) contains supplementary material, which is available to authorized users.

## Background

Primary Sjögren’s syndrome (pSS) is a systemic autoimmune disease characterized by infiltration of exocrine glands by T and B lymphocytes that, by producing chemokines and cytokines, play a fundamental role in coordinating the chronic inflammatory process [1, 2]. Different subsets of effector T cells, including Th1, Th2, Th17, regulatory T cells, and follicular helper T cells, have been demonstrated to participate in the pathogenesis of pSS [3–5]. In particular, follicular T-helper cells seem to be relevant biomarkers of pSS [6] and cytokines such as interleukin (IL)-21 and IL-23 play a major role in their modulation [6, 7]. Although the aberrant activation of the T cell response observed in pSS has partially been explained by the interaction of T lymphocytes both with the ductal epithelium and the infiltrating B cells via costimulatory molecules (i.e., CD40/CD40L) and the BAFF axis, other immunological mechanisms could play a crucial role.

Autophagy is a lysosome-mediated catabolic process that allows cells to degrade damaged organelles and misfolded proteins, and to recycle nutrients [8]. During autophagy, portions of cytoplasm are sequestered by double-membrane vesicles, the autophagosomes, and degraded after fusion with lysosomes for subsequent recycling. Autophagy is a genetically regulated process that requires the activity of autophagy-related gene (Atg) proteins [9]. Atg5 plays a central role in the autophagic process by binding to Atg12, acting as a dimeric complex together with Atg16, inducing the curvature of the phagophore. Atg7 acts like an E1 ubiquitin-activating enzyme [10]. The Atg5-Atg12 complex contributes to the conjugation of m﻿icrotubule-associated﻿ protein 1 light-chain 3 (MAP1LC3, also known as﻿﻿ LC3, the homolog of Atg8 in yeast) to the lipid phosphatidylethanolamine. The unlipidated cytosolic form of LC3 is called LC3-I, whereas the lipidated form, referred to as LC3-II, is localized into the autophagosomal membranes throughout the maturation process of the autophagosome, therefore being considered an autophagic marker [11]. Other different genes and proteins finely regulate the autophagy process, among them: ATG16L1, the main component of a large protein complex essential for autophagy; the immunity-associated GTPase family M (IRGM), which regulates autophagy formation in response to intracellular pathogens; ATG5, a ubiquitin ligase essential for autophagosomal elongation; HSPA8/HSC70, a protein that binds to nascent polypeptides to facilitate correct protein folding; HSP90AA1, involved in the proper folding of specific target proteins; p62/SQSTM1, a protein that links ubiquitinated proteins to the autophagic machinery to enable their degradation [12].

Double-membrane autophagosomes have been identified in both human and murine T lymphocytes [13, 14], and a crucial role for autophagy in T lymphocyte development, survival, and proliferation has been observed [15, 16]. Moreover, aberrant regulation of autophagy has been implicated in an increasing number of autoimmune disorders such as systemic lupus erythematous (SLE), rheumatoid arthritis, ankylosing spondylitis, multiple sclerosis, Crohn disease, and vitiligo [17–19]. To date, only a few studies have been able to evaluate the potential role of autophagy in pSS induction and development. In animal models, it has been shown how activation of the autophagosomal pathway is involved in preventing or alleviating salivary and lacrimal gland dysfunctions [20–22]. More recently, Katsiougiannis et al. [23] demonstrated that, in human salivary glands (HSG) cells, endoplasmic reticulum (ER) stress induced autophagy and apoptosis in both patients with SS and controls. However, all the studies focused on the possible role of the autophagy pathway in salivary gland epithelial cells and there is no evidence of its effect on T cells.

In this study, we investigated the autophagy process in human T lymphocytes from salivary gland tissues and peripheral blood of patients with pSS, hypothesizing a possible role of autophagy in the increasing survival of autoreactive T cells. We also assessed the possible relationship between the level of T lymphocyte autophagy and the clinical features of pSS. Evidence provided in this study strongly supports a prominent role for CD4^+^ T lymphocyte autophagy in the local immune processes in salivary glands of patients affected by pSS.

## Methods

### Patients and samples

Thirty consecutive patients affected by pSS attending the Sjögren Clinic of Sapienza University of Rome were enrolled in this study. Patients were classified as pSS according to the American-European Consensus Group (AECG) criteria [24]. The presence of other underlying autoimmune diseases and/or hepatitis C virus infection was carefully excluded. Patients were characterized for the presence of extraglandular manifestations such as arthralgia/arthritis, cryoglobulinemia, Raynaud’s phenomenon, and pulmonary, renal, neuro-muscular, or pancreatic involvement. All pSS patients underwent Schirmer-I, tear break up time and ocular dye (fluorescein and lissamine green) tests to assess the presence of keratoconjunctivitis sicca. Disease activity and damage were evaluated according to Sjögren’s syndrome disease activity index (SSDAI), EULAR Sjögren’s syndrome disease activity index (ESSDAI), and Sjögren’s syndrome disease damage index (SSDDI) [25, 26]. Minor salivary glands were excised through the mucosa of the lower lip. Salivary gland biopsies taken for histology and gene analysis were divided in equal parts. One part was embedded in paraffin for immunohistochemistry (IHC) and the other snap frozen in RNAlater solution for real-time polymerase chain reaction (RT-PCR) analysis. Hematoxylin and eosin stained tissue sections were evaluated for the presence of focal and periductal lymphocytic infiltration with the lymphocyte focus score (FS), determined on the basis of the number of inflammatory cell aggregates containing >50 lymphocytes per 4 mm^2^ [27]. Steroids or hydroxychloroquine were discontinued at least 24 h before venipuncture. Sera were collected and frozen at −70 °C until used.

Thirty healthy control (HC) subjects and twenty patients with sicca syndrome or nonspecific chronic sialoadenitis (nSS) not satisfying AECG criteria for pSS, sex- and age-matched, were included as control groups for peripheral blood and tissue sample analyses, respectively.

Informed consent was obtained from all participants and the local ethics committee approved the study (Comitato Etico dell’Università Sapienza di Roma, Prot. 1882/15).

### Serology

Antinuclear antibodies were evaluated by indirect immunofluorescence (IIF); anti-SSA/Ro and anti-SSB/La antibodies of IgG isotype were measured by commercial enzyme-linked immunosorbent assay (ELISA; Diamedix, Miami, FL, USA). Rheumatoid factor (RF) was detected using an immunonephelometry test (Behring, Marburg, Germany). Serum IL-23 and IL-21 levels were quantified by ELISA (Biolegend, San Diego, CA, USA), following the manufacturer’s instructions.

### Cell purification

Peripheral blood mononuclear cells (PBMCs) were isolated by Ficoll-Hypaque (Cedarlane, Euroclone, Pero, Milan, Italy) density-gradient centrifugation. Separation of untouched T cells from PBMCs was performed by immunomagnetic-based depletion of non-T cells using the Pan T Cell isolation Kit II (MiltenyiBiotec, Bergisch-Gladbach, Germany). Purity of isolated cells, assessed by flow cytometer, routinely reached at least 97%.

### Flow cytometry

Surface phenotyping of freshly isolated PBMCs was performed with combinations of monoclonal antibodies (mAbs) conjugated with fluorescein isothiocyanate (FITC), phycoerythrin (PE), peridinin chlorophyll protein (PerCP), or allophycocyanin (APC) as described previously [18]. Conjugated mAbs against human CD3, CD4, CD8, CD45RA, CD62L, CD95, HLA-DR, and control mouse IgG1 (all from BD Biosciences, San Josè, CA, USA) were used. The naive T cell subset was defined as CD45RA^+^CD62L^+^, whereas the remaining cells comprised the memory subsets (CD45RA^−^CD62L^+^, central memory subset; CD45RA^+^CD62L^−^ and CD45RA^−^CD62L^−^, effector memory subset) [28]. Acquisition was performed on a FACSCalibur cytometer (BD Biosciences) and 50,000 events per sample were run. Data were analyzed using the Cell Quest Pro software (BD Biosciences).

### Sodium dodecyl sulphate-polyacrylamide gel electrophoresis (SDS-PAGE) and Western blot

To evaluate whether autophagy was detectable in freshly isolated peripheral T lymphocytes of pSS patients, we examined the expression of an established set of autophagosomal markers: LC3-II, p62/SQSTM1, and Atg5 [29]. Briefly, purified T lymphocytes were lysed in lysis buffer (100 mM Tris HCl, pH 8; 150 mM NaCl; 1% Triton X-100; 1 mM MgCl_2_; 25 mM Na_3_VO_4_) in the presence of complete protease inhibitor mixture (Sigma-Aldrich, Milan, Italy). Protein content was determined by the Bradford assay (Bio-Rad Laboratories, Segrate, Milan, Italy). The samples were loaded onto 15% SDS-PAGE and, after electrophoresis, proteins were transferred onto nitrocellulose membrane (Amersham Hybond-ECL; GE Healthcare Europe, Munich, Germany) by means of a Trans-Blot transfer cell (Bio-Rad Laboratories). The membranes were then blocked in TBS containing 0.05 Tween 20 ﻿(TBS-T) and 5% skimmed milk for 1 h at room temperature, rinsed, and incubated with the intended antibodies (Abs) in TBS-T and 5% bovine serum albumin (BSA). Rabbit anti-human LC3, anti-p62/SQSTM1, and anti-Atg5 (Cell Signaling Technology, Beverly, MA, USA) Abs were used as primary antibodies. Excess primary Ab was removed by washing the membrane in TBS-T. The membranes were then incubated with peroxidase-conjugated anti-rabbit IgG Ab (Bio-Rad Laboratories) and the reaction was developed using SuperSignal West Pico Chemiluminescent Substrate (Pierce, Thermo Fisher Scientific, Waltham, MA, USA). To ensure the presence of equal amounts of protein, the membranes were reprobed with a rabbit anti-human β-actin Ab (Amersham, Gent, Belgium). Quantification of protein expression was performed by densitometry analysis of the autoradiograms (GS-700 Imaging Densitometer; Bio-Rad Laboratories) [18].

### mRNA extraction and quantitative TaqMan RT-PCR gene expression on salivary gland biopsies

Labial salivary biopsies from patients and controls, immediately after removal, were stored in RNAlater solution (Applied Biosystems, Foster City, California, USA) and processed, as previously described [30]. Samples were run in triplicate using the Step-One Real-Time PCR system (Applied Biosystems). Relative quantification of gene expression between controls and patients were determined using the ΔΔCt method, as previously described [30]. Final values were expressed as relative quantification.

### Immunohistochemistry (﻿IHC)

Formaldehyde-fixed, paraffin-embedded tissue samples were obtained from minor labial salivary gland biopsies of all 30 patients with histologically proven pSS and from 20 patients with nSS as controls. All samples were obtained after informed consent during routine diagnostic procedures. IHC analysis was performed on 3-μm thick paraffin-embedded sections, as previously described [31]. In most pSS patients and controls, it was possible to analyze multiple biopsies taken at the same time.

The organizational level of lymphocytic infiltrates in minor salivary glands was graded by staining of serial sections using B lymphocytes, T lymphocytes, and follicular dendritic cell markers. The histological evaluation of the number of foci, the degree of organization of the mononuclear aggregates, and the presence of germinal centers (GC) were assessed on the basis of the histologic appearance and confirmed by CD3, CD20, and CD21 staining by two blinded observers (FC and GG) [32]. Abs directed against CD20 (Monoclonal Mouse Anti-Human CD20, 1:100 dilution; Dako, Glostrup, Denmark), CD21 (Monoclonal Mouse Anti-Human CD21, 1:100 dilution; Dako), CD3 (Polyclonal Rabbit Anti-Human CD3, 1:100 dilution; Dako), CD4 (Monoclonal Mouse Anti-Human CD4, 1:100 dilution; Dako), CD8 (Monoclonal Mouse Anti-Human CD8, 1:100 dilution; Dako), LC3-II (Rabbit polyclonal anti-Human LC3B, 1:250 dilution; Abcam, Cambridge, UK), and Atg5 (rabbit monoclonal anti-human ATG5, 1:100 dilution; Abcam Cambridge, UK) were used, as previously described [32].

### Indirect Immunofluorescence﻿ (IIF) and confocal analysis

IIF analysis was performed on paraffin-embedded sections in order to characterize LC3-II-expressing cells and to assess whether LC3-II colocalized with Atg5, CD4, and CD8 cells. Sections were incubated with anti-human-LC3 (Novus Biologicals, Littleton, CO, USA), anti-Atg5 (Novus Biologicals), anti-CD3, anti-CD4, and anti-CD8 Abs (Dako) and then labeled with FITC- or Rhodamine Red-conjugated anti-mouse or anti-rabbit Abs plus RNasi (200 ng/mL) and counterstained using Toto-3 iodide (642/660; Invitrogen, Monza MB, Italy). Confocal analysis was used to acquire fluorescence staining.

### Statistical analysis

Results were analyzed on SP-SS (version 14.0). The Mann–Whitney unpaired test was used to compare quantitative variables in different groups. Spearman’s rank correlation coefficient was applied for calculation of the correlation between parallel variables in single samples. Linear regression analysis was used to display a best fit line to the data. *p* values <0.05 were considered as significant.

## Results

### Clinical, histologic, and immunophenotypic characteristics of patients with pSS

The demographic, clinical, and serological characteristics of the enrolled patients are summarized in Table 1.

**Table 1:** Demographic, clinical, and serological characteristics of the patients and controls

	Primary Sjögren’s syndrome (*n* = 30)	Nonspecific chronic sialoadenitis (*n* = 20)	Healthy controls (*n* = 30)
Age, mean years (range)	59.3 (32–75)	55 (29–77)	50 (25–68)
Sex, female/male	29/1	18/2	25/5
Disease duration, mean years (range)	5.4 (1–26)	–	–
Xerophtalmia, *n* (%)	29 (96.6)	12 (60)	0
Xerostomia, *n* (%)	27 (90)	12 (60)	0
Parotid enlargement, *n* (%)	11 (36.6)	0	0
Arthralgia/arthritis, *n* (%)	23 (76.6)	10 (50)	0
Cryoglobulinemia, *n* (%)	1 (3.3)	0	0
Raynaud’s phenomenon, *n* (%)	6 (20)	12 (60)	0
Pulmonary involvement, *n* (%)	1 (3.3)	0	0
Myositis, *n* (%)	2 (6.6)	0	0
Pancreatic involvement, *n* (%)	1 (3.3)	0	0
Lymphoma, *n* (%)	1 (3.3)	0	0
Antinuclear antibodies, *n* (%)	28 (93.3)	5 (25)	0
Anti-SSA/Ro, *n* (%)	17 (56.6)	0	0
Anti-SSB/La, *n* (%)	13 (43.3)	0	0
Rheumatoid factor, *n* (%)	11 (36.6)	2 (10)	0
IL21, pg/mL median (range)	292 (164–839)	310 (150–999)	301 (177–777)
IL23, pg/mL median (range)	2113 (1838–3567)	2050 (1200–5600)	1913 (1361–10936)
CSS, *n* (%)	6 (20)	–	–
Hydroxychloroquine, *n* (%)	16 (53.3)	–	–
SSDAI, median (range)	3 (0–8)	–	–
ESSDAI, median (range)	5.1 (0–24)	–	–
SSDDI, median (range)	1.7 (1–7)	–	–

*ESSDAI* EULAR Sjögren’s syndrome disease activity index, *SSDAI* Sjögren’s syndrome disease activity index, *SSDDI* Sjögren’s syndrome disease damage index

All pSS patients had a FS ≥1. Nine pSS patients (30%) had GCs determined on IHC evaluation of tissue sections.

**Fig. 1:**
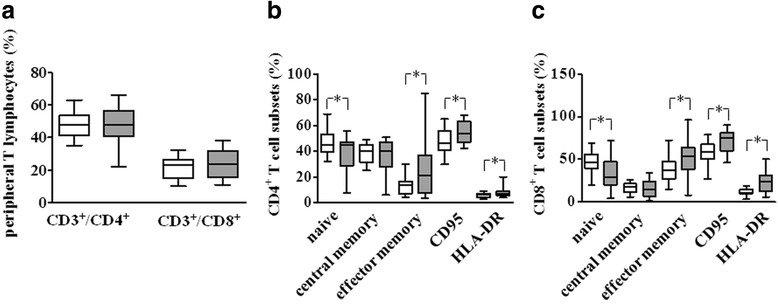
Flow cytometry analysis of distribution of T cell subsets from pSS patients and from HC. For CD4^+^ and CD8^+^ T lymphocyte subsets, data are expressed as the percentage of each subset within the CD4^+^ or CD8^+^ population considered as 100%. Data are represented as box plots (*white* and *grey* box plots for HC and pSS patients, respectively) displaying medians, 25th, and 75th percentiles as boxes, and 10th and 90th percentiles as whiskers. **a** Peripheral T lymphocytes (%). **b** CD4^+^ T cell subset (%) vs HC. **c** CD8^+^ T cell subset (%). *p < 0.05 vs HC

The peripheral distribution of the main T cell subpopulations was evaluated by flow cytometry (Fig. 1a–c). As shown in Fig. 1b and c, the percentage of naïve T cells was significantly reduced, whereas the percentage of effector memory T cells was significantly higher in pSS patients, as compared to HC in CD4^+^ (*p* = 0.02 for both naïve and memory T cells) and CD8^+^ (*p* = 0.02 and *p* = 0.04 for naïve and memory T cells, respectively) T cells. A significantly higher percentage of CD95- and HLA-DR-positive cells was also detected in CD4^+^ (*p* = 0.01 and *p* = 0.04, respectively) and CD8^+^ (*p* = 0.003 and *p* = 0.005, respectively) T cell subsets (Fig. 1b and c). These findings suggest a general state of activation within circulating T cells in the disease population. Nevertheless, the altered distribution of lymphocyte subsets was independent of disease activity, organ manifestation, and treatment.

### Autophagy levels in freshly isolated T cells from pSS patients

**Fig. 2:**
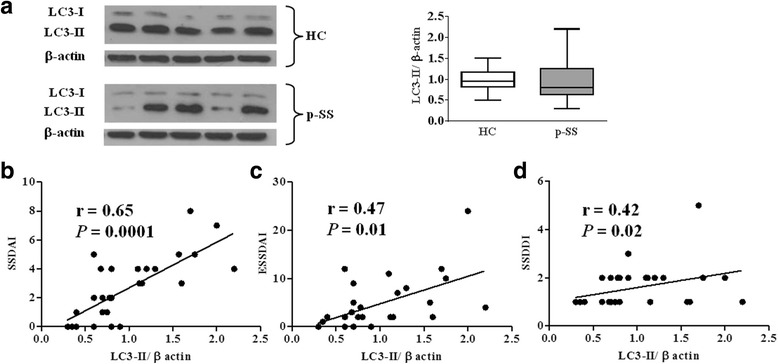
Western blot analysis of the autophagy marker LC3-II in freshly isolated T cells from pSS patients and from HC, and correlation between LC3-II levels and clinical index scores. **a** Blots shown are representative of independent experiments carried out in T cells from healthy controls (HC; *n* = 30) and from primary Sjögren’s syndrome (pSS) patients (*n* = 30). Quantification of LC3-II levels relative to β-actin in normal and pSS T cells is also shown. Data are represented as box plots (*white* and *grey* box plots for HC and pSS patients, respectively) displaying medians, 25th, and 75th percentiles as boxes, and 10th and 90th percentiles as whiskers. **b–d** LC3-II levels positively correlate with SSDAI, ESSDAI, and SSDDI scores. The rho (*r*) and *p* values were determined using the Spearman’s rank correlation analysis. *Solid lines* represent best fits as estimated by linear regression analysis. *ESSDAI* EULAR Sjögren’s syndrome disease activity index, *SSDAI* Sjögren’s syndrome disease activity index, *SSDDI* Sjögren’s syndrome disease damage index

As shown in Fig. 2a, a great interindividual variability for LC3-II levels was present in peripheral T lymphocytes from pSS patients and no significant differences in the level of LC3-II were found between patients and HC. Similarly, there were no significant differences between lymphocytes from patients and those from HC in the levels of p62/SQSTM1, and Atg5 (data not shown). Interestingly, LC3-II levels showed a significant positive correlation with SSDAI (*r* = 0.65, *p* = 0.0001), ESSDAI (*r* = 0.47, *p* = 0.01), and SSDDI (*r* = 0.42, *p* = 0.02) scores (Fig. 2b–d). Since some patients with pSS were treated with hydroxychloroquine (see Table 1) and this drug was demonstrated to be a potent inhibitor of autophagy [29], we also evaluated our results in light of bias. However, no significant difference was found between hydroxychloroquine-treated and untreated patients.

### Autophagy-related genes are upregulated in the salivary glands of pSS patients and correlate with IL-23p19 and IL-21 expression levels

**Fig. 3:**
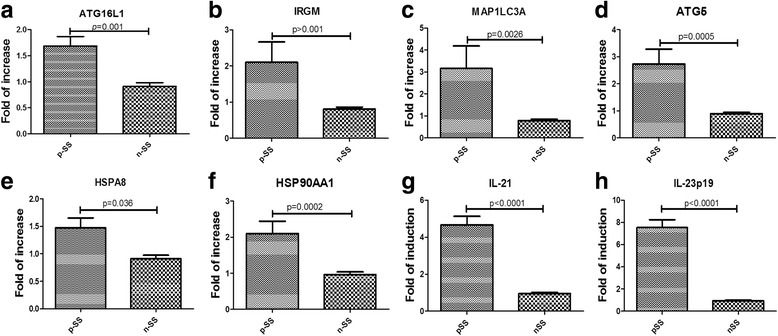
Autophagy and IL-21/IL-23 mRNA gene expression in pSS salivary glands. **a**–**f** Relative mRNA quantification of autophagy genes was assessed by quantitative RT-PCR in minor salivary glands samples obtained from primary Sjögren’s syndrome (pSS) patients and nonspecific chronic sialoadenitis (nSS). Data are expressed as mean + SEM. **g, h** Upregulation of IL-23p19 and IL-21 mRNA in salivary glands of pSS patients but not in nSS

We evaluated the expression of *atg* genes in the salivary glands of pSS patients by RT-PCR. As shown in Fig. 3, a significant upregulation of mRNA specific for *ATG16L1* (Fig. 3a), *IRGM* (Fig. 3b), *MAP1LC3A* (Fig. 3c), and the Atg5 protein (Fig. 3d) was observed in pSS compared to nSS. Interestingly, mRNA specific for *HSPA8/HSC70* (Fig. 3e) and for *HSP90AA1* (Fig. 3f) were also significantly upregulated in the salivary glands of all pSS patients compared to nSS.

Furthermore, a significant upregulation of IL-21 and IL-23p19 mRNA in salivary glands of pSS patients, but not in nSS, was observed (Fig. 3g and h). IL-23p19 and IL-21 mRNA levels were directly and significantly correlated with the mRNA levels of *ATG16L1*, *ATG5*, and *IRGM* autophagy genes (Additional file 1: Figure S1). On the contrary, no differences were observed in serum levels of IL-23 and IL-21 between patients and controls (Table 1).

### Characterization of the tissue expression of autophagy proteins in salivary glands by IHC, IIF, and confocal analysis

**Fig. 4:**
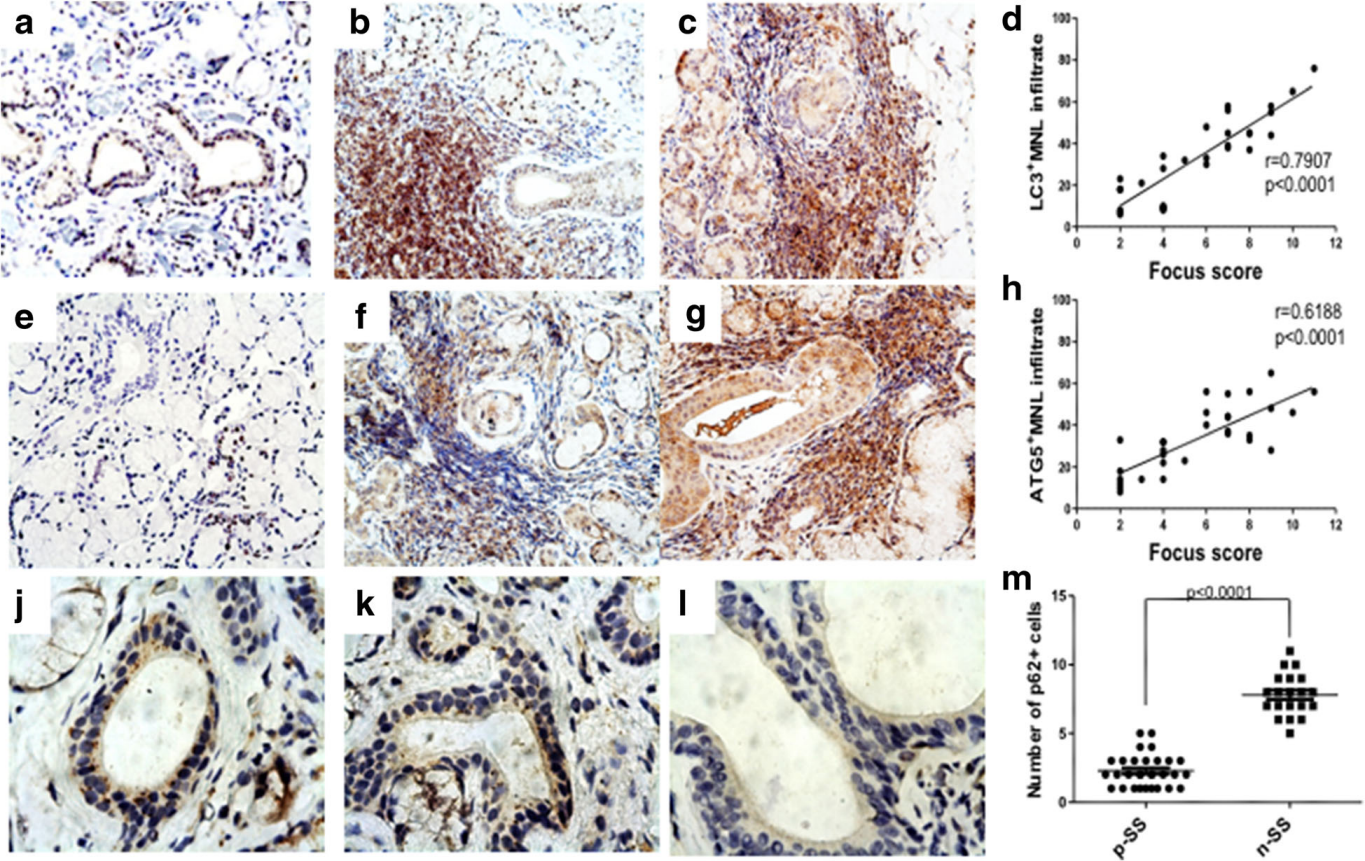
IHC evaluation of autophagy expression in minor salivary gland biopsies of patients with primary Sjögren’s syndrome (pSS) and nonspecific chronic sialoadenitis (nSS). **a**–**c** Representative photomicrographs showing 3-μm thick paraffin-embedded sections of lip gland biopsy specimens obtained from nSS (**a**) and patients with pSS (**b, c**), stained for LC3-II. **d** Correlation of LC3-II with FS. LC3-II expression in infiltrating cells by IHC was correlated with the FS of pSS. **e**–**g**. Representative photomicrographs showing 3-μm thick paraffin-embedded sections of lip gland biopsy specimens obtained from nSS (**e**) and patients with pSS (**f, g**), stained for Atg5. **h**. Atg5 expression in infiltrating cells by IHC was correlated with the FS of pSS. **j**–**l**. p62/SQSTM1 immunoreactivity in lip gland biopsies of nSS (**j, k**) and pSS patients (**l**). **m** Number of p62/SQSTM1 cells in pSS and nSS. (**a**–**c**, **e**–**g**, original magnification × 250; **j**–**l** original magnification × 400)

After demonstrating the strong upregulation of genes involved in the autophagy pathway, in order to localize Atg5- and LC3-II-expressing cells, we performed IHC and IIF. As shown in Fig. 4, a strong and diffuse expression of LC3-II (Fig. 4b and c) and Atg5 (Fig. 4f and g) was observed in the salivary glands of pSS patients compared to nSS (Fig. 4a and e). The staining was mostly localized among infiltrating inflammatory mononuclear cells and ductal epithelial cells. Moreover, the number of immunoreactive cells was significantly correlated with the degree of tissue inflammation as evaluated by FS (Fig. 4d and h). We also evaluated the expression of p62/SQSTM1 (Fig. 4j–l). Consistent with the increased activation of the autophagy pathway, p62/SQSTM1 was significantly downregulated in the salivary glands of pSS compared to nSS (*p* < 0.0001) (Fig. 4l). Interestingly, patients with GC in salivary glands also had a higher number of LC3-II- and Atg5-positive cells (data not shown). We evaluated a possible correlation between the expression (presence vs absence of the protein) of Atg5 and LC3-II and the clinical data of all our patients, and no statistically significant difference was observed. No relevant difference was found in antinuclear antibody, extractable nuclear antigen, and RF positivity between the two groups.

**Fig. 5:**
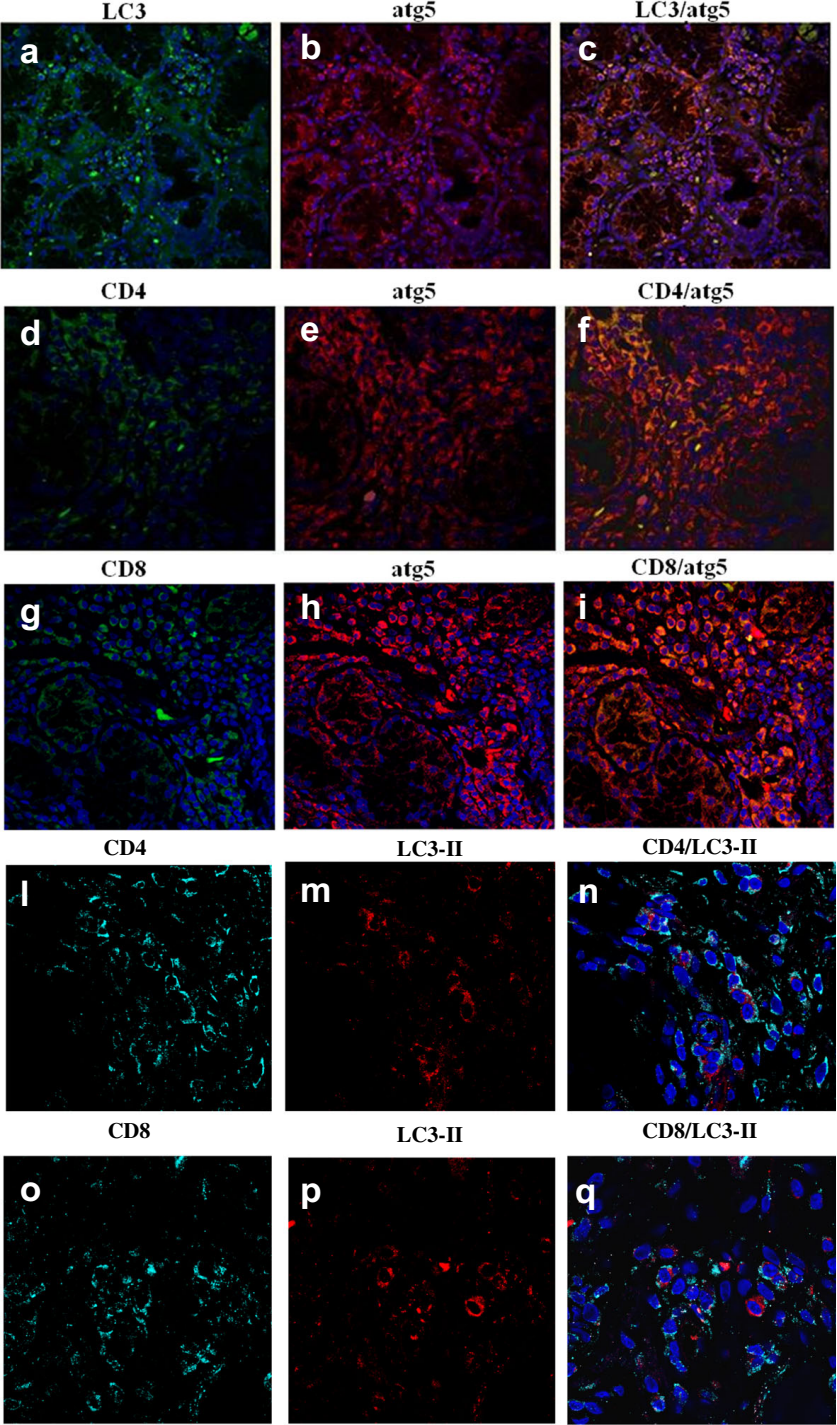
IIF on pSS salivary glands with inflammatory infiltrates evaluating LC3-II and Atg5 tissue expression in relation to CD4^+^ T cells. **a–c**. Representative image of LC3-II/Atg5 immunostainings of lip gland biopsies of pSS patients: single staining for LC3-II (*green*) (**a**) and Atg5 (*red*) (**b**), and merged double staining for LC3-II and Atg5 (**c**). **d**–**f**. Immunolocalization by confocal microscopy of CD4 (*green*) and Atg5 (*red*) in pSS salivary gland biopsies. **g–i**. Immunolocalization by confocal microscopy of CD8 (*green*) and Atg5 (*red*) in pSS salivary gland biopsies. **l–**﻿**n﻿**﻿. Immunolocalization by confocal microscopy of CD4 (*green*) and LC3-II (*red*) in pSS salivary gland biopsies. **o**–**q**. Immunolocalization by confocal microscopy of CD8 (*green*) and LC3-II (*red*) in pSS salivary gland biopsies. Significant colocalization of Atg5 with CD4 (**f**) but not CD8 (**i**), and significant colocalization of LC3-II with CD4 (**o**) but not CD8 (**q**) was detected in the inflamed glands of pSS. (**a**–**q**, original magnification × 250)

To confirm that the immunoreactivity for LC3-II and Atg5 observed in pSS was truly associated with autophagosomes, we also examined the colocalization of LC3-II with Atg5. As shown in Fig. 5a and c, a significant colocalization of LC3-II and Atg5 was clearly detectable in the salivary glands of pSS patients, confirming the tissue autophagy activation.

Finally, in order to characterize the main cellular source of autophagy in salivary glands of pSS patients, double immunofluorescent confocal microscopy analysis was performed using mAbs for CD4, CD8, Atg5, and LC3-II (Fig. 5). Of note, in the T area, CD3^+^CD4^+^ T cells were the main cellular source of Atg5 and LC3-II in pSS salivary glands (Fig. 5d–f and l–n, respectively).

In conclusion, autophagy is increased mainly in CD4^+^ T lymphocytes from salivary glands, suggesting that the survival of these cells might initiate autoimmunity and could play a key role in pSS pathogenesis.

## Discussion

In this study, we provided the first demonstration of autophagy dysregulation in both salivary and peripheral T lymphocytes from pSS patients. A positive correlation was found between autophagy level and CD4^+^ T lymphocyte infiltration as well as tissue expression of the proinflammatory cytokines IL-21 and IL-23 in salivary glands of pSS patients. Additionally, we found that the level of autophagy in peripheral blood T lymphocytes positively correlated with pSS disease activity indexes.

pSS is a chronic systemic autoimmune disease characterized by the infiltration of T and B lymphocytes into exocrine glands [1, 2]. Evidence involves both innate and adaptive immune responses in pSS pathogenesis. The chronic inflammation provokes ductal epithelial cell death with exocrine gland dysfunction, contributing to causing xerostomia and keratoconjunctivitis sicca, typical of pSS. The upregulation of autophagy-related markers (at both the mRNA and protein levels) observed in this study in the salivary glands of pSS, mainly in CD4^+^ T cells, is intriguing considering previous studies showing that T cells, mostly CD4^+^ T cells, predominate in the salivary gland lesions and play a crucial role in the induction and/or maintenance of pSS (e.g., providing a stimulus for B lymphocytes and promoting the destruction of the gland through cytotoxicity) [33]. Growing evidence suggests that autophagy has a positive, cytoprotective, and homeostatic role in activated T cells [16] and dysregulation of this process, as herein observed, could favor the persistence of pathogenetic CD4^+^ T cells in salivary glands. For instance, autophagy supports metabolic functions of T cells at various stages of their maturation and effector function and is crucial for T cell development at the precursor stage, also regulating peptide presentation in stromal cells and professional antigen-presenting cells. Indeed, in other autoimmune diseases, and prevalently in SLE, the role of autophagy in T lymphocytes has been extensively studied: *atg5* ^−^/^−^ CD8^+^ T lymphocytes displayed increased cell death, and *atg*5^−^/^−^ CD4^+^ and CD8^+^ T cells demonstrated deficient proliferation after T cell receptor stimulation [15]; *atg7*-deficient T lymphocytes had increased cell death and also showed expanded mitochondria, expanded ER content, and altered calcium flux [33]. Similarly, *atg3*-deficent mature T cells showed defective ER homeostasis and mitochondrial clearance and elevated levels of reactive oxygen species, which might induce autophagy and consequently the apoptotic pathway [34]. Although the precise mechanisms leading to autophagic dysregulation in SLE are still not understood, this pathway has been implicated in promoting survival of both human and murine T cells [18, 35].

The role of autophagy in salivary gland homeostasis and pathology has been studied exclusively in the epithelial cells, demonstrating its prosurvival and homeostatic role [20, 21]. More recently, Katsiougiannis et al. [23] investigated the relation between ER stress and autophagy induction in salivary epithelial cells. In this study, thapsigargin was used as an autophagy inducer, but the authors speculate that, in real life, other inducers of ER stress can be responsible for this phenomenon, such as an epithelial cell viral infection or sympathetic hormone insults leading to autoimmune initiation, as reflected by autoantibody redistribution on the cell surface [23]. Therefore, further studies are needed to elucidate the role and the possible pathogenetic effect of autophagy in pSS. Our study innovatively investigates the possible pathogenetic role of autophagy in a different cell population, T cells, demonstrating that autophagy is increased in salivary glands T lymphocytes and speculating that this could promote the survival of a subset of cells that can initiate autoimmunity. In our study, the autophagy level in salivary glands was also significantly correlated with FS and damage indexes. These results are expected because patients with a higher FS or GCs in their salivary glands are more likely to have worse disease and prognosis [36, 37]. Moreover, according to the prognostic role of T cell autophagy in pSS, it would be interesting to evaluate the role of autophagy in lymphoproliferative complications in pSS. In fact, the identification of biomarkers that could be clinically valuable for making diagnoses and determining disease progression in lymphoid malignancies are currently lacking.

Previous studies have demonstrated a role for autophagy in modulating the expression of IL-23, a cytokine strongly implicated in the pathogenesis of pSS and correlated with clinical manifestations [31]. Interestingly, we found a significant correlation between the level of Atg genes and that of IL-23p19 and IL-21 in salivary glands from pSS patients. Modulation of IL-23 production by autophagy has been recently demonstrated, suggesting a role of autophagy in bridging innate and adaptive immune responses [38]. The correlation between autophagy gene expression and IL-21 in the inflamed salivary glands of pSS represents, in our opinion, an important and novel finding. Although no direct correlation between autophagy and IL-21 production has been demonstrated, the differentiation of IL-21-producing Th17 cells seems to be dependent on MHC II expression by CD11c^+^ cells [39]. In this regard, it has been recently demonstrated that pathogens and pattern-recognition receptors can directly activate autophagy to upregulate MHC II presentation [40]. In light of this evidence, the autophagy activation in pSS salivary glands might be the result of a viral or bacterial stimulation and might be involved in driving the differentiation of IL-21-producing cells. Nevertheless, in our study no difference in serum levels of IL-23 and IL-21 between patients with pSS and HC was observed. We speculate that the discrepant results in blood compared to tissue might reflect local rather than systemic cytokine modulation.

Finally, as expected, circulating T cells in the pSS population were demonstrated to have a general state of activation, given the imbalance of naïve and memory T cell distribution and the increased expression of T cell activation markers. Interestingly, despite the absence of significant differences in terms of autophagy levels between pSS and controls, a significant correlation between peripheral T lymphocyte autophagy and disease activity and damage indexes in pSS was found.

## Conclusions

This is the first study that investigates autophagy in T lymphocytes in patients with pSS. According to our results, an increase in the autophagy levels in T lymphocytes from salivary glands in pSS patients could represent the local origin of the pathogenic process in which an immune stimulus could activate the autophagic pathway and therefore promote the survival of a subset of lymphocytes. Our findings may be disease-specific or simply reflect the general increase in inflammation seen in this condition and further studies over larger cohorts of patients are mandatory to validate the possible use of T lymphocyte autophagy-related molecules as prognostic peripheral biomarkers and to evaluate the implications of autophagy as a therapeutic target in pSS.

## Additional file

Additional file 1: Figure S1. IL-23p19 and IL-21 mRNA level correlations with mRNA levels of autophagy genes. IL-23p19 and IL-21 mRNA levels were directly and significantly correlated with the mRNA levels of ATG16L1, ATG5, and IRGM autophagy genes. (TIF 50 kb)

## Abbreviations

Ab(s): Antibody(ies)
AECG: American-European Consensus Group
APC: Allophycocyanin
Atg: Autophagy-related gene
*ATG16L1*: Autophagy-related gene 16-like 1
BSA: Bovine serum albumin
ELISA: Enzyme-linked immunosorbent assay
ER: Endoplasmic reticulum
ESSDAI: EULAR Sjögren’s syndrome disease activity index
FITC: Fluorescein isothiocyanate
FS: Focus score
GC: Germinal center
HC: Healthy controls
HSG: Human salivary gland
HSPA8/HSC70: Heat shock 70 kDa protein 8/ heat shock cognate protein 70
IHC: Immunohistochemistry
IIF: Immunofluorescence
IL: Interleukin
IRGM: Immunity-associated GTPase family M
LC3: Protein 1 light-chain 3
mAbs: Monoclonal antibodies
MAP1LC3A: Microtubule-associated protein 1 light chain 3 alpha
nSS: Nonspecific chronic sialoadenitis
p62/SQSTM1: p62/sequestosome 1 protein
PBMC: Peripheral blood mononuclear cell
PE: Phycoerythrin
PerCP: Peridinin chlorophyll protein
pSS: Primary Sjögren’s syndrome
RF: Rheumatoid﻿ Factor
RT-PCR: Real-time polymerase chain reaction
SDS-PAGE: Sodium dodecyl sulphate-polyacrylamide gel electrophoresis
SLE: Systemic lupus erythematous
SSDAI: Sjögren’s syndrome disease activity index
SSDDI: Sjögren’s syndrome disease damage index
TBS-T: Tris-buffered saline containing 0.1% Tween 20
